# Indications for the Use of Sialoendoscopy in Sialolithiasis

**DOI:** 10.17691/stm2020.12.3.05

**Published:** 2020-06-28

**Authors:** K.A. Bannikova, Yu.Yu. Bosykh, V.G. Gaitova, P.G. Sysolyatin, S.P. Sysolyatin

**Affiliations:** PhD Student, Department of Surgical Dentistry and Maxillofacial Surgery; Peoples’ Friendship University of Russia, 6 Miklukho-Maklaya St., Moscow, 117198; Dental Surgeon; Center for Endoscopic Dentistry and Maxillofacial Surgery “Endostom”, 12 Proyezd Berezovoy Roshchi, Moscow, 125252;; Assistant, Department of Anatomy; I.M. Sechenov First Moscow State Medical University (Sechenov University), 8/2 Malaya Trubetskaya St., Moscow, 119991;; Dental Surgeon; Center for Endoscopic Dentistry and Maxillofacial Surgery “Endostom”, 12 Proyezd Berezovoy Roshchi, Moscow, 125252;; Professor, Department of Hospital Surgical Dentistry and Maxillofacial Surgery; Novosibirsk State Medical University, 52 Krasny Prospect, Novosibirsk, 630091; Professor, Department of Surgical Dentistry and Maxillofacial Surgery; Peoples’ Friendship University of Russia, 6 Miklukho-Maklaya St., Moscow, 117198; Director; Center for Endoscopic Dentistry and Maxillofacial Surgery “Endostom”, 12 Proyezd Berezovoy Roshchi, Moscow, 125252;

**Keywords:** sialolithiasis, sialoendoscopy, sialolith, salivary calculi, sialoscope, endoscopy, sialolith extraction, endosialoscopic assistance.

## Abstract

**The aim of the study** is to determine indications for the use of sialoendoscopy in the diagnosis and treatment of sialolithiasis.

**Materials and Methods.:**

The study involved 115 patients with sialolithiasis, who underwent cone beam computed tomography, ultrasound diagnosis of the salivary glands, and sialoendoscopy, in addition to the standard general clinical examination.

**Results.:**

Sialoendoscopy makes it possible to detect a stone, determine its shape, relative size, mobility, and assess the condition of the salivary ducts. It is impossible to obtain this information by other methods, though it is very important for treatment decision making. The design of the sialoscope and its special instruments make it possible to proceed with sialolith extraction immediately after detecting it.

**Conclusion.:**

The absolute indication for the use of sialoendoscopy is mobile calculi less than 5 mm in diameter (L1 according to F. Marchal’s LSD classification). In case of immobile sialoliths less than 4–8 mm in size, located in the main duct (L2), endoscopy should be used as a method supplementary to ductotomy. When sialoliths are located in the distal parts behind the areas of bending or stricture (L3a and L3b), the use of endoscopy is not indicated.

## Introduction

In the diagnosis of sialolithiasis, the most commonly used methods are non-contrast or contrast-enhanced projection radiography, multispiral and cone beam computed tomography (CBCT), ultrasound scanning, and other methods. However, despite such a wide range of diagnostic tools, the number of errors reaches 46% [1].

Numerous studies on the treatment of sialolithiasis show that it is adequate to remove the stone in order to restore the normal function of the salivary gland. Nevertheless, the most commonly applied treatment method is gland extirpation, since ductotomy and other stone removing techniques have no clear indications and are used “on the off chance” [2–4].

The first reports on the endoscopy of the large salivary glands appeared in the last years of the past century [5–7]. This promising approach attracted attention of medical researchers and medical equipment engineers. To date, there has been developed complex equipment consisting of a sialoendoscope and supplementary instruments, making sialoendoscopy a full-fledged medical technology. The design of modern endoscopes allows them to move along the salivary ducts, examine them, and perform visually controlled maneuvers, including grasping and extraction of sialoliths or lithotripsy [8, 9].

Sialoendoscopy has not yet become widespread, but some foreign authors consider it the most effective method for the diagnosis and treatment of sialolithiasis [10–14]. However, even strong advocates of sialoendoscopy admit that its capabilities are limited. To date, indications for the use of sialoendoscopy have not been defined [2, 9–11, 15]. This procedure is safe, but rather expensive due to the high cost of equipment and disposable tools.

In this regard, **the aim of this study** is to determine indications for the use of sialoendoscopy in the diagnosis and treatment of sialolithiasis.

## Materials and Methods

The study involved 115 patients with sialolithiasis treated at the Center for Endoscopic Dentistry and Maxillofacial Surgery “Endostom” (Moscow).

In addition to the standard general clinical examination protocol, all patients underwent mandatory CBCT, ultrasound scanning of the salivary glands, and diagnostic sialoendoscopy.

**Figure 1:**
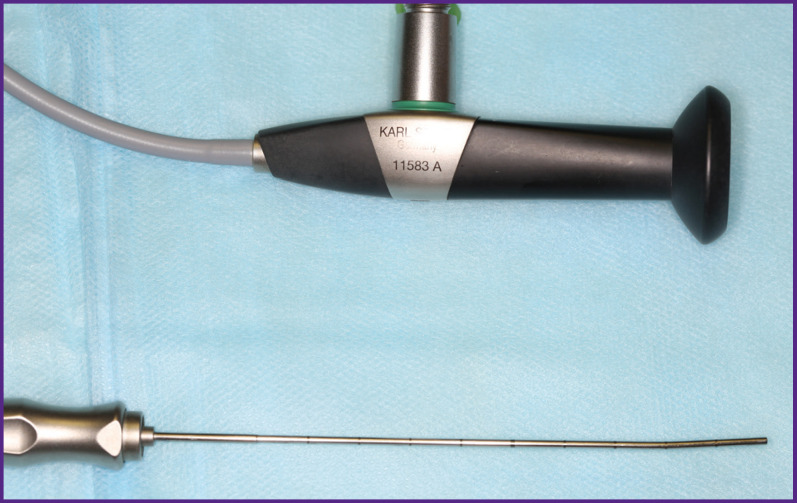
Karl Storz all-in-one sialoscope (Germany) with a diameter of 1.6 mm

Endoscopic examination was performed in the outpatient settings using Karl Storz all-in-one sialoscopes (Germany) with diameters of 1.1 and 1.6 mm (Figure 1).

During the diagnosis, we set ourselves the following tasks: confirming the presence of sialoliths, determining the exact number, size, shape, structure, location, and mobility of the stones. It was important to obtain information about the state of the duct system itself. To organize the diagnostic data, we used the LSD (Lithiasis, Stenosis, and Dilation) classification proposed by F. Marchal [3].

Diagnostic sialoscopy was performed under local anesthesia and drug sedation, which made it possible to proceed with sialolith extraction immediately after the stone was detected. Endosialoscopic micro forceps and multi-link basket grasper were used as working instruments for stone extraction.

Intraductal fragmentation of bigger sialoliths was performed using the sialoendoscopic drill and contact lithotripsy with FiberLase U2 thulium fiber laser (IPG Photonics Corporation, IRE-Polus, Russia) with a fiber diameter of 200 and 400 μm.

## Results

Comparing the informational value of ultrasound, CBCT, and sialoendoscopy in the diagnosis of sialolithiasis, we came to the conclusion that none of them provide comprehensive information. Each method has its advantages and disadvantages. For example, ultrasound requires no special training, it is the most common and inexpensive primary diagnostic method. However, in our study, it was the ultrasound examination that showed a large number of diagnostic errors due to subjective interpretation of examination results by doctors: small sialoliths with a diameter less than 1 mm were taken for cicatricial changes, while strictures and stenoses were interpreted as sialoliths.

Cone beam computed tomography proved to be an indispensable diagnostic method for detecting the presence of sialoliths in the salivary duct and identifying their exact number and size. However, this method provides no information about the ductal system of the salivary gland.

Diagnostic sialoscopy made it possible to evaluate the characteristics of sialoliths (according to the LSD classification) and the surrounding soft tissues alike. It should be noted that despite the apparent advantages of this method, a full-fledged sialoendoscopy seems to be impossible in the presence of strictures or stenoses that impede the advancement of the optics along the duct.

**Figure 2:**
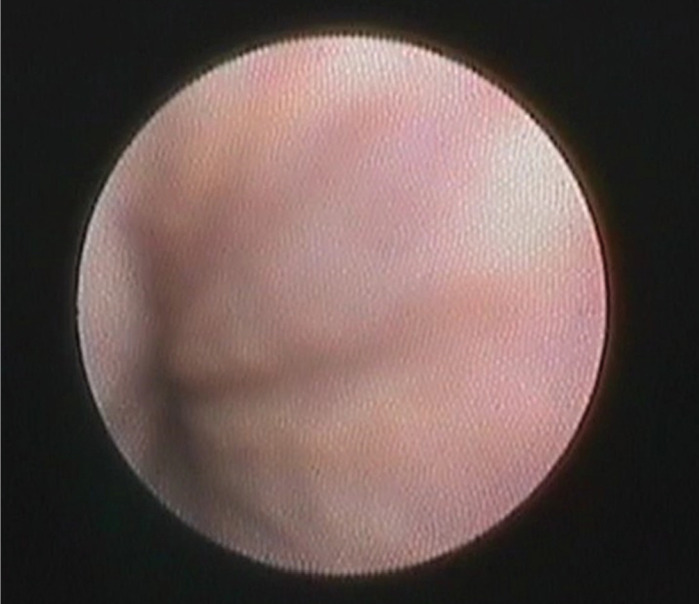
Stricture in the proximal part of the submandibular salivary duct

A total of 115 salivary glands were examined for sialolithiasis. Successful diagnostic sialoscopy of the duct was performed in 110 cases (95.6%). In 5 patients (4.4%), optics could not be advanced deeper into the duct due to the presence of stricture in the proximal part of the ductal system (Figure 2).

**Figure 3:**
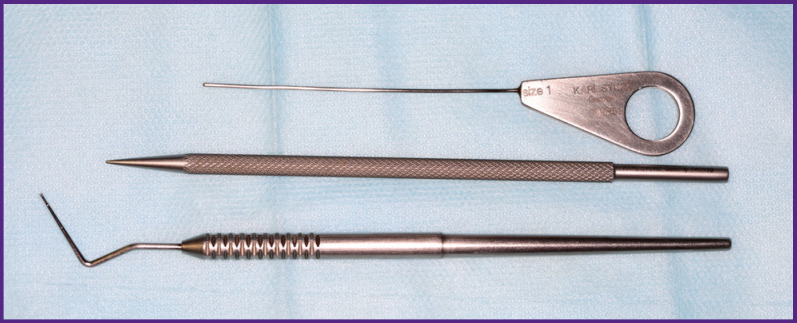
Instruments for bougieurage of the duct orifice

Optics with the external diameter of 1.1 mm was used only in ducts with cicatricial changes or severe stenoses. In all other cases, it was preferable to work with a sialoscope of 1.6 mm diameter as it was easier to control inside the duct. Besides, a set of sialoscopic instruments that allow performing medical manipulations in the salivary duct are available in a much wider range for that diameter sialoscope. Various dilators and bougies were used as general-purpose tools to dilate junctions and facilitate inserting the sialoscope through the mouth into the duct (Figure 3).

**Figure 4:**
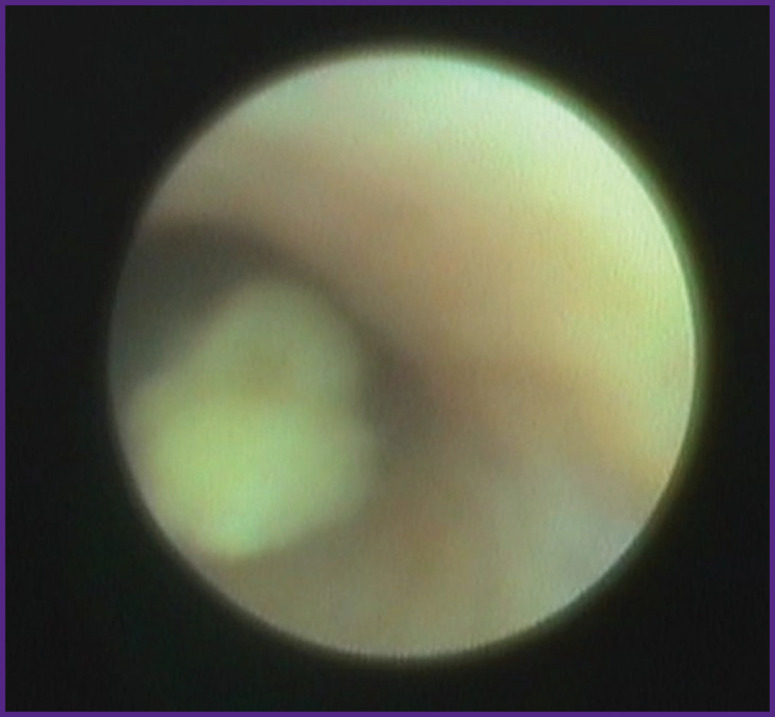
Floating sialolith

During diagnostic sialoscopy, sialoliths were detected in 110 patients (95.6%). In 52 cases (47.2%) there were smooth mobile sialoliths with the diameter less than that of the duct. Stones sized less than 2.5 mm floated freely along the duct (Figure 4), bigger stones had limited mobility within the dilated area.

**Figure 5:**
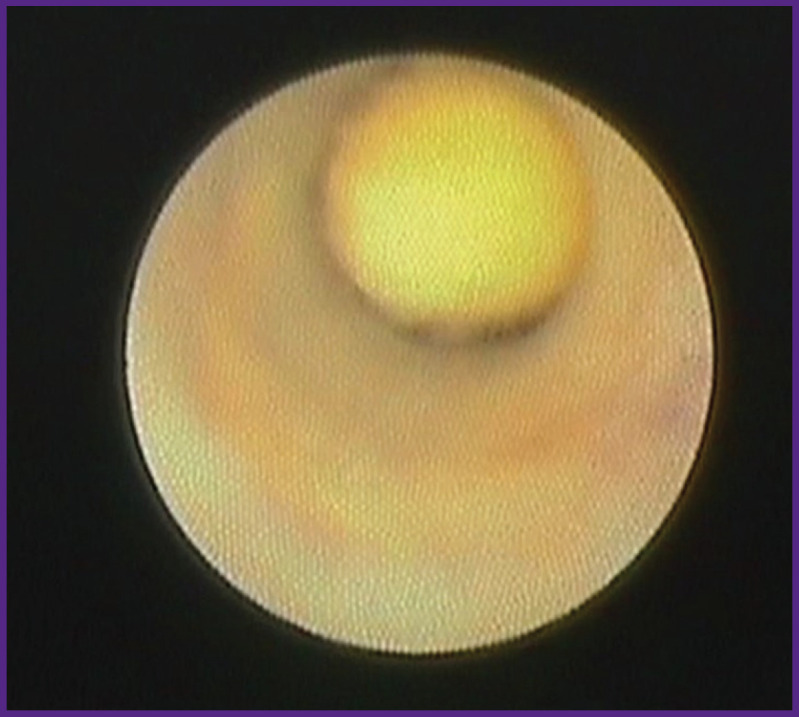
Fixed sialolith

In 29 patients (26.4%), there were intruded stones that blocked the main lumen of the duct completely, disrupting the outflow of saliva. In 18 cases, fixed adherence of sialoliths to the duct wall was caused by a large stone sized between 6 and 8 mm (Figure 5), in 11 cases — by the presence of duct stricture.

**Figure 6:**
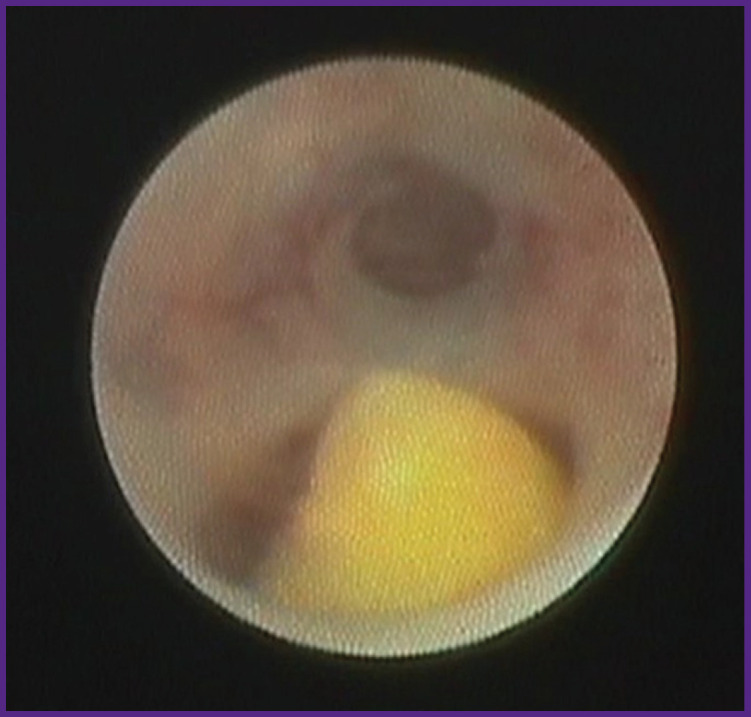
Sialolith behind the bending of the duct

In the remaining 29 patients (26.4%), sialoliths were located in the 5^th^ order ducts behind the areas with stenosis or pronounced bending (Figure 6), therefore, it was impossible to explore and evaluate them thoroughly. According to CBCT data, the diameter of such stones ranged between 5 and 31 mm. In 5 cases, according to radiodiagnosis, multiple sialolithiasis occurred. During diagnostic sialoscopy in these 29 patients, we managed to examine only the anterior stone that, as a rule, limited the advancement of optics past it into the distal portion, making it impossible to see the remaining sialoliths.

By consolidating data on sialoliths, obtained during sialoendoscopy and preliminary diagnosis data, including ultrasound examination and CBCT, we structured them according to the LSD classification offered by F. Marchal (see the Table).

**Table:** Distribution of detected sialoliths according to the LSD classification offered by F. Marchal

Sialoliths	of Number patients	Mobility	Location	Visualization
L1 (1–5 mm)	52	Yes	The main duct or parenchyma	Complete
L2 (4–8 mm)	29	No	The main duct	Complete
L3а (7–31 mm)	20	No	Parenchyma	Partial
L3b (3–5 mm)	9	No	Parenchyma	Partial

After detecting sialoliths, an attempt was made to remove them endoscopically in all cases. Sialoendoscopy and a wide range of various sialoscopic instruments enabled us not only to examine a sialolith, but also to evaluate its structure and density by probing. Such diagnostic data were important for assessing the possibility of intraductal fragmentation of large stones and subsequent endoscopic extraction of stone debris. For these purposes, there was an endosialoscopic hand drill and FiberLase U2 thulium fiber laser in our armamentarium.

Only loose sialoliths with irregular surface could be fragmented successfully with a drill, though not in all cases. The drill tended to slide off the surface of the stone, causing injury to the duct wall. The method of endoscopic fragmentation and stone removal was successful only in 3 cases (L2), though fragmentation was attempted in 28 cases.

**Figure 7:**
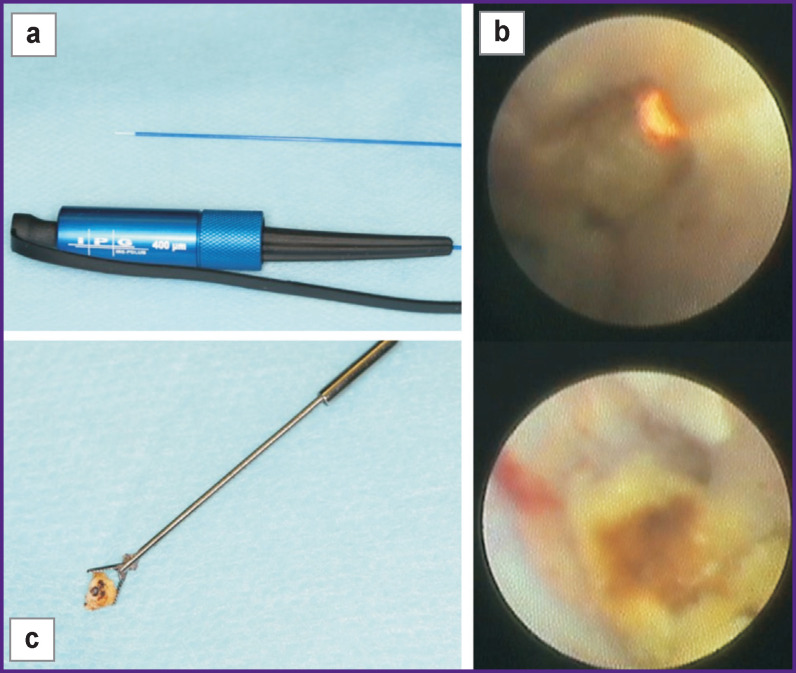
Intraductal contact lithotripsy: (a) the FiberLase U2 thulium fiber laser with a fiber diameter of 400 μm; (b) sialolith fragmentation stages; (c) extracted fragment

The FiberLase U2 thulium fiber laser was used for intraductal contact lithotripsy as an experimental modality in 5 patients (Figure 7) who had single sialoliths (L2) with a diameter up to 8 mm in the distal portion. A fiber with a diameter of 200 or 400 μm was introduced into the working channel of the sialoscope and the laser beam was focused strictly on the center of the stone under constant irrigation of the duct with physiological saline. Contact laser lithotripsy was performed directly under strict visual control. Fragmentation of sialoliths was successful in all 5 cases, regardless of the initial stone density. In 2 cases, sialoliths were immobile and located behind the bending. As a result, it was difficult to direct the fiber to the center of the stone. This led to a strong heating of the surrounding tissues, their subsequent damage, and perforation of the duct wall. Later, during the control diagnostic sialoscopy, we observed mild cicatricial deformities of the duct in the area of perforations.

Thus, the method of intraductal laser lithotripsy has great potential when used correctly and rationally, though it requires further in-depth investigation.

Micro forceps were used to retrieve small sialoliths and move them to the anterior ducts. To remove the stones, we used various flexible multi-link basket graspers with 3, 4, 5 links. Altogether, sialoliths were removed using endosialoscopic instruments in 29% (32/110) of cases. These sialoliths floated freely along the duct and were classified as L1.

In 18.2% (20/110) of patients, additional papillotomy was required as sialoliths (L1) were gripped and extracted from the duct with wire basket or forceps, but they were unlikely to pass through the duct orifice without it being cut.

In 26.4% (29/110) of cases, sialoliths were removed in the course of endoscopically assisted ductotomy. Stones sized 4–8 mm were immobile and blocked in the main duct (L2). To extract them, the endoscope was positioned directly on the stone, so ductotomy and sialolith removal were performed over the luminous tissues using translumination in the oral cavity.

In 26.4% (29/110) of patients, it appeared to be impossible to extract calculi using endoscopic techniques due to unfavorable location of stones in the distal portion of the salivary gland behind the site of stenosis or bending (L3a, L3b).

Thus, sialoendoscopy is the most informative and accurate diagnostic modality for sialolithiasis. It provides the possibility not only to detect the stones, but also to identify stone shape and relative size, mobility, the general state of the ducts, particularly, in the stone location area, etc. It is impossible to obtain this information by other methods, though it is extremely important for treatment decision making. At the same time, sialoendoscopy is limited or even impossible in the presence of strictures or duct stenosis. Therefore, we recommend using CT and ultrasound for the primary diagnosis of sialolithiasis, while diagnostic sialoscopy should be used as the ultimate diagnostic procedure determining the choice of sialolith removal method.

## Conclusion

Sialoendoscopy is highly effective for the extraction of mobile sialoliths with a diameter of less than 5 mm, classified as L1 according to F. Marchal’s classification.

In presence of immobile calculi sized 4–8 mm, located in the main duct (L2), endoscopy should be used only as a supplementary method to ductotomy.

In cases of sialoliths located in the distal parts behind the areas of bending or stricture (L3a and L3b), the use of endoscopy is not indicated.

Laser lithotripsy is likely to expand the possibilities of sialoendoscopic treatment significantly, but it requires further investigation, clinical observations, and evaluation of long-term effects.
